# Severe Clinical Outcomes of Tuberculosis in Kharkiv Region, Ukraine, Are Associated with Beijing Strains of *Mycobacterium tuberculosis*

**DOI:** 10.3390/pathogens8020075

**Published:** 2019-06-10

**Authors:** Olha Konstantynovska, Mariia Rekrotchuk, Ivan Hrek, Anton Rohozhyn, Nataliia Rudova, Petro Poteiko, Anton Gerilovych, Eric Bortz, Oleksii Solodiankin

**Affiliations:** 1 Kharkiv Medical Academy of Postgraduate Education, 61176 Kharkiv, Ukraine; seolkah@gmail.com (O.K.); grek.ivan.md@gmail.com (I.H.); pekin2006@ukr.net (A.R.); petrosantonenko@gmail.com (P.P.); 2 National Scientific Center Institute of Experimental and Clinical Veterinary Medicine (NSC IECVM), 61023 Kharkiv, Ukraine; vakulenkovmv@gmail.com (M.R.); rudovanatawa@ukr.net (N.R.); antger2011@gmail.com (A.G.); 3 Department of Biological Sciences, University of Alaska Anchorage, Anchorage, AK 99508, USA

**Keywords:** TB, pulmonary tuberculosis, *Mycobacterium tuberculosis*, MIRU-VNTR genotype, Beijing strain, risk factors, strain replacement, superinfection, re-infection, hospital setting, TB/HIV co-infection

## Abstract

Genotypic variation in Beijing lineages of *Mycobacterium tuberculosis* (MTB), the causative agent of tuberculosis (TB), has been associated with hyper virulence and the spread of extensively and multiple drug (X/MDR) resistant MTB strains in Eastern Europe, Central Asia, and East Asia. The clinical outcomes of 215 new cases of TB among the population of the Kharkiv region of Eastern Ukraine were analyzed to uncover factors associated with severe infection. Infecting MTB strains were profiled by 5 locus exact tandem repeats (ETRs) and 15 locus mycobacterial interspersed repetitive unit-variable number tandem repeat (MIRU-VNTR) genotyping. Among diverse MTB genotypes discovered in Ukraine, the Beijing genotype (MIRU-VNTR 42425) was significantly associated with risk factors for severe outcomes of disease in the study population, including TB/HIV co-infection and treatment failure. Strain replacement (superinfection) was observed in 10 patients, suggesting repeated exposure to novel MTB strains in hospital or community settings. Inclusion of MTB genotyping data may identify at-risk patients and improve treatment adherence to prevent X/MDR development for effective public health response against tuberculosis in Ukraine.

## 1. Introduction

Despite the considerable effort of the global strategy to combat tuberculosis (TB), conducted under the auspices of the World Health Organization (WHO) and other organizations, TB remains a burdensome problem among infectious diseases throughout the world. Annually, about 10 million new cases of TB are registered [1]. A significant danger has been recognized in the territory of Ukraine with the spread of drug resistant forms of TB. Ukraine is among the five countries with the highest burden of multidrug-resistant tuberculosis (MDR-TB) [1]. The situation for patients with tuberculosis (TB), especially multi- and extensively drug-resistant (M/XDR) TB, has dramatically worsened in Ukraine because of the military conflict that started in 2014 in eastern Ukraine. The European Centers for Disease Prevention and Control reported 3522 patients with M/XDR-TB living in Ukraine in 2012, compared to 1421 in all European Union/European Economic Area countries [2].

Among the several genotypic lineages of *M. tuberculosis* (MTB) that have been identified in the territory of Ukraine, the Beijing genotype is particularly interesting. According to recent studies, increased survival and reproductive capacity in human macrophages has been demonstrated, as well as high transmissibility of this genotype [3]. MTB Beijing strains have also been associated with hyper virulence and drug resistance, with resistance mediated by single nucleotide polymorphisms (SNP) and large gene deletions (e.g., resistance to isoniazid) [4,5]. However, in other countries, genomic variation has been found to be natural for this lineage and not necessarily associated with drug resistance [6]. MTB Beijing also induced severe pro-inflammatory neutrophil-dominated infiltration into necrotic lesions in the lungs of a mouse model of TB [7].

The Beijing genotype of *M. tuberculosis* was initially identified in a geographical region concentrated in the northeast of China, Korea and Japan more than 6600 years ago. The worldwide spread of the MTB Beijing genotype occurred in several stages, driven by the Industrial Revolution, the First and Second World Wars, and the emergence of HIV infection [8]. One hypothesis posits that workers of the Chino-East Railway brought the Beijing genotype to the territory of the Soviet Union from China. The pathogen spread widely among prisoners of the GULAG and among the civilian population. At present, the Beijing genotypes have widely circulated in prisons in Russia and the countries of the former USSR, thus representing a reservoir for the generation of multi-drug resistance and for the spreading of diverse MTB genotypes to neighboring countries [9,10,11]. In this regard, the eastern part of Ukraine and the Kharkiv region, which directly borders Russia (where one-fourth of Beijing genotype isolates belongs to the B0/W148 group [6]), is a natural transportation route for the expansion of MTB Beijing strains to Europe.

Since Ukraine is one of the countries with the greatest burden of tuberculosis, and multidrug-resistant forms in particular, there is a natural concern about the emergence and development of superinfection by *M. tuberculosis*. It was shown that patients could be simultaneously infected with two strains of *M. tuberculosis* [12]. Sometimes a more stable strain replaces, displaces, or suppresses a less stable strain, leading to superinfection, a phenomenon observed in several bacterial infections [13]. In addition, two different strains can coexist simultaneously in the patient’s body and a mixed-infection can develop. Superinfection has been shown to play a decisive role in the activation of passive carriers of tuberculosis and has a significant impact on anti-tuberculosis treatment, with treatment outcomes worse in patients infected with both resistant and sensitive strains [11,14]. Superinfection has also been found in cases of heteroresistance to isoniazid and rifampin, where MTB with differing drug resistance profiles infects an individual patient and has been associated with poor treatment outcomes in countries of the former USSR [11,15]. In this regard, it is important to distinguish between the reactivation of old tuberculosis foci or granulomas in the lungs and the emergence of superinfections or mixed infections. This is necessary to determine the most effective therapeutic approaches for managing and treating patients with drug resistant strains, to positively influence disease outcomes, and to improve the state of epidemiological control and biosafety in medical institutions.

The aim of this study was to analyze the outcomes of 215 new cases of TB identified in the population of Kharkiv Region, Ukraine, and to find any associations between the strain genotype using exact tandem repeats (ETR) and mycobacterial interspersed repetitive unit-variable number tandem repeat (MIRU-VNTR)-profiles of MTB, and risk factors and outcomes of TB treatment. We found that among diverse MTB genotypes in Ukraine, the Beijing genotype (MIRU-VNTR 42425) was associated with risk factors and severe disease outcomes.

## 2. Results

### 2.1. Dominant Genotypes of M. tuberculosis in TB Cases in Kharkiv Region, Ukraine

In 2015–2016, we analyzed 215 new cases of pulmonary TB in tuberculosis hospital units in the Kharkiv region in eastern Ukraine. Among the 215 patients, 80% were men (*n* = 196) and 20% were women (*n* = 19). The age of the patients ranged from 23 to 84 years (average age 49 ± 1.2 years). Patients were observed by clinical, radiological, and laboratory examination; clinical findings in all cases included in this study were severe pulmonary tuberculosis with massive MTB spreading, which was confirmed by culture and smear-positivity.

**Figure 1:**
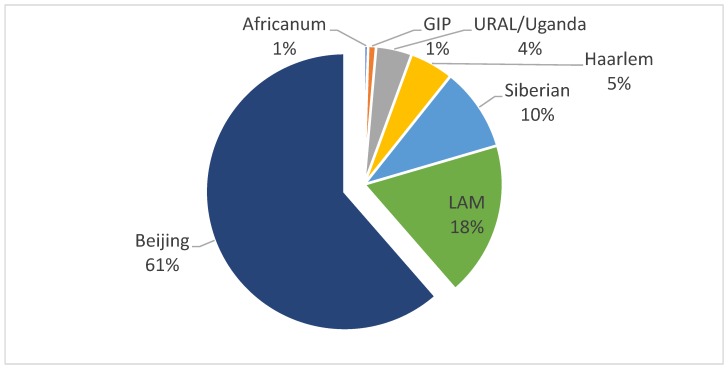
Genotypes of *M. tuberculosis* among 215 severe hospitalized cases of pulmonary tuberculosis in Kharkiv region, Ukraine (2015–2016). Severe *tuberculosis* (TB) cases were identified and culture, and *Mycobacterium tuberculosis* (MTB) genotyped by 5 locus exact tandem repeats (ETR), and 15 locus mycobacterial interspersed repetitive unit-variable number tandem repeat (MIRU-VNTR) methods as described in text. *LAM*, Latin American-Mediterranean; individual genotypes (*GIP*), unique individual genotypes.

MTB genotypes were identified in the patient cohort by standardized polymerase chain reaction (PCR)-based ETR and MIRU-VNTR genotyping. Among the 215 patients with pulmonary TB, we found that 131 patients (61%) were infected with MTB Beijing strains, by 5 locus ETR genotyping (Figure 1). The remaining 84 (39%) patients were infected with other MTB genotypes: Latin American-Mediterranean (39 patients, 18%), Siberian (21 patients, 15%), Haarlem (11 patients, 5%), URAL/Uganda (9 patients, 4%), or other strains (Figure 1). Among four strains, which could not be assigned by ETR genotyping, two (2) were identified using 15 loci MIRU-VNTR genotyping as lipoarabinmannan LAM and two (2) as individual genotypes (GIP) that resemble the Haarlem strain phylogenetically. Both of these GIP cases were isolated from patients who live in small villages of the Kharkiv region. In contrast to the rapid spread of Beijing strains of MTB in eastern Europe, these two isolates may be unique to the Kharkiv region of Ukraine.

**Figure 2:**
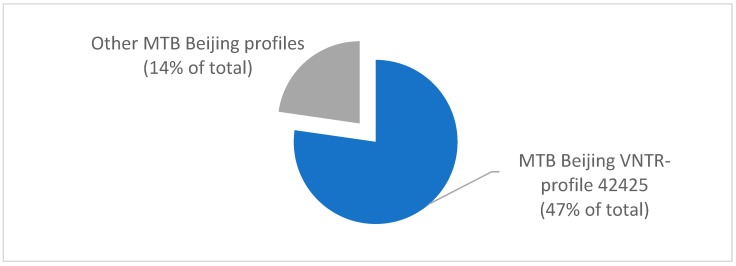
Genotypic profiling of 131 *M. tuberculosis* Beijing genotypes by MIRU-VNTR genotyping at 15 loci for cases in the Kharkiv region, Ukraine (2015–2016). Profile 42425 represented 105/131 MTB Beijing cases.

To better understand the genotypic distribution of the predominant MTB Beijing strains, additional MIRU-VNTR genotyping at 15 loci were performed. Of 131 patients, 105 (78%) were infected with MTB Beijing strain with MIRU-VNTR genotype profile 42425, representing 47% of all TB cases in this study. In contrast, 29 (22%) were infected with other MTB Beijing strains, representing 14% of the total TB cases (Figure 2). For a subset of MTB Beijing strains, more detailed genotypic identification was performed using MIRU-VNTR plus genotyping at 24 loci [16]. The results of this confirmatory study did not identify additional genotypic variations beyond those identified in 15 locus MIRU-VNTR (Figure 2) and 5 locus ETR (Figure 1) genotyping (data not shown).

### 2.2. Analysis of Risk Factors for Severe TB Outcomes

Next, we analyzed risk factors for clinical progression and outcomes of MTB strains, in relation to the dominant Beijing genotypes. Rate analyses demonstrated a significant statistical difference in socio-medical risk factors for the group of patients infected with the Beijing 42425 strain, compared to the group of patients infected with other strains (Table 1). The treatment adherence rate (leading to outcomes of cure or treatment completion, by WHO guidelines [17]) was significantly lower for patients with the 42425 strain (45% to 61%; F = 5.53, *p* = 0.0198), while other risk factors showed significantly higher association for patients with the Beijing 42425 strain over other strains, namely: 57% compared to 41% for alcoholism (F = 5.65, *p* = 0.02), 19% compared to 6% for drug addiction (F = 8.13, *p* = 0.004), and 17% compared to 4% for HIV co-infection (F = 9.33, *p* = 0.0019). Other potential risk factors in TB (age, sex, ex-imprisonment, educational attainment, non-infectious comorbidities of the heart and vessels, nervous and digestive systems, adverse reactions to medications, volume of lung tissue damage, and intoxication syndrome) did not yield significant statistical associations among groups infected with the MTB Beijing genotype 42425 and other strains.

### 2.3. Effectiveness of Treatment

We examined treatment effectiveness in relation to MTB genotypes. Treatment outcomes were for a pulmonary TB patient with laboratory-confirmed TB at the beginning of treatment. Patients who adhered to treatment experienced positive outcomes where the patient was effectively cured (a series of negative diagnostic tests) or completed treatment (treated but without a negative evidentiary follow-up test), according to WHO guidelines defining TB treatment outcomes [17]. Other outcomes included failure of treatment, lost to follow-up, and death, showing the spectrum of negative outcomes in this study population (Table 2). For positive outcomes, the treatment success rate (cured) was found to be significantly lower for patients infected with the Beijing 42425 strain (11% compared to 25%; F = 5.16, *p* = 0.0323), and the treatment completed rate was also lower (13% compared to 34%; F = 13.79, *p* = 0.0151) for the MTB Beijing 42425 infection (Table 2). The unsuccessful treatment rate for the 42425 strain was higher (30% compared to 16%; F = 6.44, *p* = 0.0098), and the lethal outcome rate was higher too (33% compared to 19%; F = 6.18, *p* = 0.0198). The overall treatment effectiveness (cured plus treatment completed; i.e., positive outcomes) for patients with 42425 strain was significantly lower than for other strains (24% compared to 56%; F = 29.69, *p* = 0.0001).

### 2.4. Tuberculosis Co-Infection and Strain Replacement

The results reveal the usefulness of MIRU-VNTR typing in the context of MDR TB diagnostics. Moreover, we found a number of cases where Beijing 42425 was a super-infecting strain, or replaced by superinfection. In the study population, 9.5% of in-patients (10 out of 105) had several different genotypes, based on an analysis of sputum smears, which were examined in the course of clinical treatment process for TB (Table 3). In seven out of ten cases, genotype replacement was detected after one month, where a typically virulent MTB Beijing or LAM strain replaced another strain (LAM and Beijing replace URAL in three cases, Haarlem in three cases, as well as each other in three cases). Although virulence was not directly measured statistically in this study, strain replacement was typically followed by drug toxicity that led to variations in efficacy of treatment (increasing or decreasing efficacy).

## 3. Discussion

Our results suggest a high prevalence of the MTB Beijing genotype among TB patients in the Kharkiv region, which was associated with poor treatment outcomes. Moreover, strain replacement was observed, using MIRU-VNTR genotyping. While the clinical efficacy of particular anti-tuberculosis drug regimens and drug resistance profiles of MTB strains were not a subject of this study, the predominance of one MTB Beijing genotype (42425) illustrates the need for and utility of thorough genetic and phenotypic characterization of severe TB cases. ETR and MIRU-VNTR genotyping is a critical methodology for uncovering potential severity in an infection and genotyping MTB in geographic regions of high strain diversity and emerging drug resistance [18]. The Beijing complex, of which the MTB strain MIRU-VNTR 42425 is an example, has been associated with severe outcomes, extrapulmonary infection [19], and hypervirulence [20]. This strain complex is also highly prevalent in extensively and multiple drug (X/MDR) TB cases, suggesting development of resistance may be associated with the pathogenic characteristics, immune evasion, and aggressive nature of the infection [21,22,23,24].

In this study, we observed that severe outcomes of pulmonary TB infection in TB patients in the Kharkiv region were associated with MIRU-VNTR genotype Beijing 42425. In these patients, this strain was significantly associated with a number of risk factors: HIV positive status, drug addiction, and alcoholism (Table 1). A well-studied phenomenon, it appeared that TB/HIV co-infection increased the risk of severe outcomes. This is common in communities with a high HIV and TB burden and has been observed in settings where the Beijing genotype complex is one of the most prevalent MTB strain complexes [25]. We also found treatment adherence was a statistically significant risk factor (*p* = 0.0198, Fisher’s angular transformation). Given the limitations of this study specifically variabilities in drug usage and treatment regimens, it is not clear that a particular regimen is less or more effective against Beijing strains. Indeed, development of drug resistance may be exacerbated in a strained healthcare setting with a limited number of alternative compounds available—as in the Kharkiv region, Ukraine—over the course of this study (the endpoint was 2015–2016). The risk of treatment failure and death were significantly associated with Beijing genotype complex infection (Table 2). Poor outcomes were likely due to the aggressive clinical profile of the Beijing complex, TB, and development of drug resistance (data not shown) observed in the patients [26,27], even possibly X/MDR TB [28].

We also observed significant strain replacement, most likely due to exposure in the hospital setting or in the community (Table 3). In 7/10 cases, strain replacement, or superinfection, was observed after only 1 month of treatment, suggesting exposure in hospital settings. Severe TB, in this clinical setting, typically required directly-observed treatment and a short hospital stay. However, community and/or hospital-acquired superinfection by a MTB strain different than the primary infection has been observed, elsewhere in Eastern Europe [11]. The population in Kharkiv, Ukraine, is partly mobile, and has both highly urbanized as well as rural districts. In addition, since 2014, an influx of internally displaced persons from the conflict (ATO) zones in the nearby eastern provinces of Ukraine had disrupted MTB treatment adherence in the incoming population. This disruption potentially introduced new strains and drug resistance patterns in the resident population of Kharkiv and other regions in Ukraine proper. This population displacement-driven dynamic has been observed in the HIV/AIDS genotype diversity in Ukraine [27] and in TB in Viet Nam [28] and is under further investigation for public health response against TB.

Due to the potential for the development of drug resistance, and the risk of re-infection in hospital settings, it is important to provide early genotyping of *M. tuberculosis* for effective treatment and TB eradication. In addition, genotyping and genomics analyses form the foundation for further genotyping studies of MTB strains circulating among at-risk populations in Ukraine. Particularly, both MIRU-VNTR genotyping, and recent advances in rapid clinical laboratory genotyping and whole genome sequencing of MTB using nanopore sequencing technology [21] (currently being adopted in Ukraine), can provide a highly pertinent clinical diagnostic capability to determine the best treatment options for TB patients with risk factors and strain replacement modality. The analysis, herein, identifies Beijing complex genotypes infection in combination with TB/HIV co-infection, drug addiction, and treatment adherence, as a particular threat among the myriad of risk factors for severe TB, and will inform needed changes in the National Tuberculosis Program in Ukraine for treatment of X/MDR TB strains, as well as epidemiological detection, socio-economic factor analysis, and the public health control of virulent TB.

## 4. Materials and Methods

### 4.1. Tuberculosis Cases, Clinical Data and Exclusion Criteria

For this study, samples and clinical outcomes data from 215 new cases of severe pulmonary TB diagnosed in 2015–2016 were collected from tuberculosis hospital units in the Kharkiv region (Kharkiv oblast), Ukraine, and analyzed. Clinical criteria focused on understanding infection by MTB genotypes in patients without previous evidence of infection. The following clinical criteria were the used for study inclusion: new case of TB, absence of previous episodes of TB treatment, presence of MTB in sputum (by culture), compensation of comorbidity pathology (diabetes mellitus, chronic obstructive pulmonary disease (COPD), arterial hypertension, ischemic heart disease, HIV (CD4 not less than 200 cells per per cubic millimeter of blood). Exclusion criteria were: relapse of TB, previous episodes of TB treatment (even brief), decompensation of comorbidities, and absence of MTB in sputum by culture. All patients were observed by clinical, radiological, and laboratory examination in accordance with Order № 620 of the Ministry of Health of Ukraine [3]. The bioethical review, human subjects consent, and protection of private personal health information were conducted by the Institutional Review Board (IRB) and Ethics Commission of the Kharkiv Medical Academy of Postgraduate Education under protocol #4 for tuberculosis (TB) clinical investigation in human patients (22 September 2016), with results analyzed in compliance with Order № 620 of the Ministry of Health of Ukraine [3].

Outcomes of TB were analyzed after the continuation phase of treatment, where a clinical outcome was determined (typically after 6–24 months). This included the following clinical outcomes: cured, treatment completed, treatment failed, lost to follow-up, and death. As discussed, TB outcomes were defined according to WHO classification [29].

### 4.2. Drug Susceptibility Testing, ETR and MIRU-VNTR Genotyping

MTB identification and drug susceptibility testing (DST) were performed as recommended by the WHO. Cultures of MTB, obtained from sputum of patients by cultivation on the Lowenstein–Jensen medium, were collected in 1.5 mL test tubes with saline solution, followed by pathogen inactivation by high temperature (50 °C) and guanidine thyocionate (6 M), to adhere to biosafety practices while maintaining a reliable PCR test regimen. DNA was extracted using a sorbent method. PCR were carried out by Thermo Scientific Maxima Hot Start Green PCR kit in a 25 µL reaction volume, including 12.5 µL of MasterMixb, 1 µL of each primer, 5.5 µL of free water, and 5 µL of DNA. Initial strain genotyping was performed by using sets of primers for amplification of five (5) exact tandem repeats (ETR) loci (A, B, C, D, E). More detailed genotypic identification for the MTB Beijing strains was performed using MIRU-VNTR plus genotyping [30] with PCR at 15 standard loci using the following system of primers (Table 4). The primer annealing temperatures were analysed, and the 15 primer pairs were divided into three primer pools with annealing temperatures of 52 °C, 60 °C, and 62 °C, respectively, according to Table 4. The PCR amplification procedure used the following parameters: initial denaturation at 95 °C for 5 min, 40 cycles of 95 °C for 15 s, then annealing temperatures (see at Table 4) for 30 s and elongation at 72 °C for 30 s, followed by a final extension at 72 °C for 5 min. The formulas (Table 4) were used for VNTR determination. MTB MIRU-VNTR genotypes profiles were identified by comparison to the MIRU-VNTR plus database (http://www.MIRU-VNTRplus.org) [30].

For a subset of MTB Beijing strains, more detailed genotypic identification was performed using MIRU-VNTR plus database (http://www.MIRU-VNTRplus.org) [16] at 24 standard loci, to confirm the data obtained from 15 locus MIRU-VNTR and 5 locus ETR genotyping. The results of this confirmatory study did not identify additional genotypic variations (data not shown).

### 4.3. Biostatistical Analysis

Fisher’s angular transformation was used to calculate the statistical significance of differences in treatment outcomes, vis-as-vis the MIRU-VNTR genotyping of strains. Statistical analysis was performed using «STATISTICA^®^ for Windows 6.0» (Stat Soft Inc., Tibco, Palo Alto CA № SC14RYMMEC0001). The statistical significance level was set to *p* < 0.05.

## Figures and Tables

**Table 1:** Social and medical risk factors for MTB Beijing genotype 42425 infection.

	Strain 42425, *n* = 102		Other Strains, *n* = 113		F	*p*
**Associated Factors**	*n*	%	*n*	%		
Treatment Adherence	46	45%	69	61%	5.53	0.0198
Alcoholism	58	57%	46	41%	5.65	0.0200
Drug Addiction	19	19%	7	6%	8.13	0.0040
HIV Positive Status	17	17%	5	4%	9.33	0.0019

* F—Fisher’s angular transformation, *p*—probability value, *n*—number of patients, %—percent out of the total number. *Adherence*, treatment adherence (cured or completed treatment).

**Table 2:** Effectiveness of treatment outcomes for patients infected with MTB Beijing strain 42425.

	Strain 42425, *n* = 102		Other Strains, *n* = 113		F	*p*
**Outcome**	n	%	n	%		
Cured	11	11%	25	22%	5.16	0.0323
Treatment Completed	13	13%	38	34%	13.79	0.0151
Treatment Failed	31	30%	18	16%	6.44	0.0098
Lost to Follow-up	13	13%	11	10%	0.49	0.4900
Death	34	33%	21	19%	6.18	0.0198
Overall Treatment Effectiveness	24	21%	63	56%	29.69	0.0001

* F—Fisher’s angular transformation, *p*—probability value, n—number of patients, %—percent out of the total number. See text for definitions of treatment outcomes derived from WHO ref. [17].

**Table 3:** Strain replacement in patients with multiple genotypes of *M. tuberculosis* over the course of treatment.

	Before Treatment	After 1 Month of Treatment	After 3 Months of Treatment	After 8 Months of Treatment
1	URAL (52423)	LAM (42522)	LAM (42522)	LAM (42522)
2	LAM (32221)	LAM (32221)	LAM (42522)	LAM (42522)
3	Haarlem (22525)	LAM (42522)	LAM (42522)	LAM (42522)
4	Haarlem (31423)	Beijing (42425)	Beijing (42425)	Beijing (42425)
5	LAM (22423)	LAM (22423)	LAM (22222)	LAM (22222)
6	URAL (52423)	Beijing (42425)	Beijing (42425)	Beijing (42425)
7	Beijing (42425)	LAM (22222)	Beijing (42425)	Beijing (42425)
8	Beijing (42425)	Beijing (42425)	LAM (22222)	LAM (22222)
9	URAL (52423)	LAM (22222)	LAM (22222)	LAM (22222)
10	Haarlem (31423)	Beijing (42425)	Beijing (42425)	Beijing (42425)

**Table 4:** The system of primers used for 15 loci MIRU-VNTR genotyping.

Locus	Type	Primer Sequence	t	Formula
MIRU26	*F*	TAGGTCTACCGTCGAAATCTGTGAC	60 °C	(n-234)/51
	R	CATAGGCGACCAGGCGAATAG		
MIRU40	*F*	GGGTTGCTGGATGACAACGTGT	60°C	(n-354)/54
	R	GGGTGATCTCGGCGAAATCAGATA		
MIRU10	*F*	GTTCTTGACCAACTGCAGTCGTCC	62 °C	(n-484)/53
	R	GCCACCTTGGTGATCAGCTACCT		
MIRU16	*F*	TCGGTGATCGGGTCCAGTCCAAGTA	62 °C	(n-565)/53
	R	CCCGTCGTGCAGCCCTGGTAC		
Mtub04	*F*	CTTGGCCGGCATCAAGCGCATTATT	62 °C	(n-537)/51
	R	GGCAGCAGAGCCCGGGATTCTTC		
Mtub30	*F*	CTTGAAGCCCCGGTCTCATCTGT	62 °C	(n-247)/58
	R	ACTTGAACCCCCACGCCCATTAGTA		
Mtub39	*F*	CGGTGGAGGCGATGAACGTCTTC	62 °C	(n-272)/58
	R	TAGAGCGGCACGGGGGAAAGCTTAG		
QUB4156	*F*	TGACCACGGATTGCTCTAGT	52 °C	(n-563)/59
	R	GCCGGCGTCCATGTT		
Mtub39	*F*	CGGTGGAGGCGATGAACGTCTTC	62 °C	(n-272)/58
	R	TAGAGCGGCACGGGGGAAAGCTTAG		
QUB11b	*F*	CGTAAGGGGGATGCGGGAAATAGG	52 °C	(n-67)/69
	R	CGAAGTGAATGGTGGCAT		
Mtub21	*F*	AGATCCCAGTTGTCGTCGTC	52 °C	(n-92)/57
	R	CAACATCGCCTGGTTCTGTA		
QUB26	*F*	AACGCTCAGCTGTCGGAT	62 °C	(*n*-153)/111
	R	CGGCCGTGCCGGCCAGGTCCTTCCCGAT		

*F*—forward primer, R—reverse primer, n—the amplicon length at numbers of nucleotide pairs, t—annealing temperature. Three standard ETR Locus primer sets used in MIRU-VNTR genotyping are not shown see Ref. [16,30].
